# Conceptualising evidence-informed policymaking in European adult learning and education—Moving the intellectual agenda forward

**DOI:** 10.1007/s40955-026-00346-3

**Published:** 2026-05-11

**Authors:** Ellen Boeren

**Affiliations:** University of Glasgow, Glasgow, UK

**Keywords:** Evidence-informed policymaking, Adult learning and education, Lifelong learning, Europe, Evidenzbasierte Politik, Erwachsenenbildung, Lebenslanges Lernen, Europa

## Abstract

This paper aims to advance the conceptualisation of evidence-informed policymaking in European Adult Learning and Education. Evidence-informed policymaking is advocated for by international organisations such as the European Commission and the OECD but has also been critiqued in the academic literature. My contribution to the conceptualisation of evidence-informed policymaking in adult learning and education explicitly moves away from a simplistic linear approach in which the production and outputs of research are solely communicated to research users. Instead, I argue for an integrated model that incorporates the relational nature between various actors in policymaking. It furthermore interrogates the role of systems approaches to evidence-informed policymaking in terms of investments in leadership, governance, resources and infrastructures. I aim to position and conceptualise evidence-informed policymaking within broader discussions about evidence and policymaking against the international organisations’ ambition to increase European Member States’ participation in adult learning and education.

## Abstract (de)

Dieser Beitrag zielt darauf ab, die Konzeptualisierung evidenzbasierter Politikgestaltung in der europäischen Erwachsenenbildung voranzutreiben. Evidenzbasierte Politikgestaltung wird von internationalen Organisationen, wie der Europäischen Kommission und der OECD, befürwortet, ist jedoch in der wissenschaftlichen Literatur auch kritisiert worden. Mein Beitrag zur Konzeptualisierung evidenzbasierter Politikgestaltung in der Erwachsenenbildung distanziert sich ausdrücklich von einem linearen Ansatz, bei dem Forschungsergebnisse und -outputs ausschließlich an die Nutzenden der Forschung weitergegeben werden. Stattdessen plädiere ich für ein integriertes Modell, das die relationale Natur der Beziehungen zwischen verschiedenen Akteuren in der Politik einbezieht. Darüber hinaus hinterfrage ich die Rolle von Systemansätzen für evidenzbasierte Politikgestaltung im Hinblick auf Investitionen in Führung, Governance, Ressourcen und Infrastrukturen. Mit meinem Beitrag ziele ich darauf ab, evidenzbasierte Politikgestaltung in breiteren Diskussionen über Evidenz und Politikgestaltung vor dem Hintergrund des Bestrebens internationaler Organisationen, die Beteiligung der europäischen Mitgliedstaaten an der Erwachsenenbildung zu erhöhen, zu verorten und zu konzeptualisieren.

## Introduction: The need for adult learning and education policies

Investments in education tend to be frontloaded in people’s lives (Schuller and Watson 2009; Boeren 2016; OECD 2025a). However, rapid changes in society require ongoing skills development from cradle to grave. Advances in technology and transformations in demographic structures accelerate economic and societal challenges. Additionally, they have knock-on effects on skills needed in the labour market and in daily life (Ioannidou and Parma 2022; Bertoni et al. 2024; Jansen et al. 2025; Ordóñez de Pablos et al. 2023). Such issues are frequently pointed out by international organisations, for example in the recent OECD report ‘Getting Skills Right—Trends in Adult Learning: New Data from the 2023 Survey of Adult Skills’, part of PIAAC, the Programme for the International Assessment of Competencies. Based on such findings, they argue that the need for more effective and efficient policymaking in Adult Learning and Education (ALE) is urgent (OECD 2025a). The OECD report highlights that training during adulthood tends to be short-term and compliance based, with a significant proportion of participation going to compulsory workplace interventions such as Health and Safety training. While such courses are important, there is a need to accelerate the engagement with training interventions that equip adults with the rapidly changing demands of society, leading to transferable skills (Nägele and Stalder 2016; Fleming et al. 2021). The latest data from the Eurostat Adult Education Survey revealed that participation rates in ALE largely remain below national targets (Boeren and Kalenda 2025). Measured through a 12 months’ reference period in 2022 among adults aged 25 to 64, the average European Union participation rate sat below 40% (Eurostat 2026). This is lower than the benchmark of 47%, which was supposed to be reached by 2025, and significantly lower than the benchmark of 60% to be achieved by 2030. Participation rates tend to be highest in Nordic European countries, with considerably lower rates among several Southern and Eastern European Member States. They also significantly vary between adults from different socio-economic and socio-demographic groups (OECD 2025a; Eurostat 2026).

Within the academic literature, scholars have critiqued the increasingly instrumental and technical focus on adult learning, prioritising skills for employability rather than for personal development or active citizenship (Elfert and Rasmussen 2024; Mikulec 2026). Such debates have engaged with shifts in ALE policy priorities from UNESCO’s traditional humanistic focus to ALE for economic competitiveness and growth as the leading themes in OECD and European Commission reports. This shift has led to the growth of ‘governance by numbers’ approaches to monitor engagement with ALE and its potential outcomes (Ball 2018; Grek 2020). Critiques are underpinned by the notion that education tends to be normative and political, and that its direction should be influenced by democratic processes rather than effectiveness measures. For example, Biesta (2007) argued that moral questions about the aims of education remain essential, rather than solely relying on ‘what works’ in terms of reaching instrumental education outcomes. Such critiques have been ongoing in recent years (Baek et al. 2024; Biesta 2026; Milana and Tarozzi 2021).

Broadly speaking, regardless of scholars’ or international organisations’ views on the purposes of ALE, the academic and policy-oriented literature asks for more attention to ALE policymaking. This is needed to recognise the role of ALE as part of a lifelong learning approach to education, extending learning beyond post-initial settings. Given these considerations, the study of policy issues constitutes its own stream of research within the wider field of ALE. To date, scholars have drawn on a range of theories to investigate the processes behind the formation of adult learning policies. Examples include the application of historical institutionalism (Desjardins and Kalenda 2025), the use of political economy frameworks (Desjardins 2017; Desjardins and Ioannidou 2020) and a focus on stakeholder influence within policy networks (Milana et al. 2020; Torlone 2023). One approach to policymaking that has received plenty of attention in domains such as health, but only to a lesser and more recent extent in education, is ‘evidence-informed policymaking’ (EIPM). In recent years, the OECD’s Centre for Educational Research and Innovation (CERI) undertook the ‘Strengthening the Impact of Education Research’ project, leading to a suite of reports and policy briefings such as ‘Yes Minister, Yes Evidence’ (OECD 2024), ‘Who Really Cares about Using Education Research in Policy and Practice’ (OECD 2023) and ‘Everybody Cares about Using Education Research Sometime: Perspectives of Knowledge Intermediaries’ (OECD 2025a). Funding streams like the European Commission’s Horizon Europe framework have called for projects to better understand the role of evidence in education policymaking in education (European Commission 2026). However, to date, research studies on this topic remain limited, especially in the field of ALE. Given increased attention to EIPM by the leading organisations, it is the aim of this paper to further understand the nature of EIPM, specifically in the policy domain of ALE.

## Aims

This paper advances scholarly debates on the conceptualisation of EIPM in ALE, making an original and conceptual contribution to its research agenda. It engages with critiques of EIPM as constructive feedback to advance conceptual thinking rather than discarding them. Firstly, the paper engages with broader conceptual debates on the terms ‘evidence’ and ‘evidence use’. Secondly, it reviews existing EIPM frameworks, tracing its evolution from simplistic ‘linear’ to more ‘integrative’ conceptualisations (Best and Holmes 2010). Thirdly, it unpacks such integrative conceptualisation to better understand its various dimensions and relationships between them. Fourthly, I uncover the complexities of EIPM in relation to the European Commission’s policy ambitions to increase ALE participation rates across its Member States. Fifthly, I then build a ‘heuristic device’ that offers an integrated conceptualisation of EIPM’s preconditions to advance participation in ALE in the context of European policies. This ‘device’ is not meant to be prescriptive on the operationalisation of EIPM in the context of European ALE. Rather, this paper aims to provide a key conceptual reference point to the intellectual agenda on a policy approach currently under-researched in European ALE policy studies. I end this paper by providing recommendations for future research to expand such intellectual agenda.

## Conceptual debates on ‘evidence’ and ‘evidence use’

### Evidence

A focus on EIPM requires, in the first place, an explanation of what is meant by the term ‘evidence’. The Oxford Dictionary defines evidence as ‘the facts, signs or objects that make you believe that something is true’ (OED 2026). The literature presents debates on the ‘sources’ of evidence versus the ‘quality’ of such evidence. What counts as ‘evidence’ remains contested within the literature. In its narrow sense, evidence refers to findings of systematic research, often undertaken in an academic context (MacKillop and Downe 2023). However, broader definitions incorporate other types of evidence such as publicly available information on websites, expert advice, insights from citizen consultations, or tacit knowledge generated through personal experience (Klose 2024). Applied to education, such broader framing reflects on Biesta’s critique that an over-focus on evidence comes at the expense of professional teacher judgement (Biesta 2007). Rather, a more holistic interpretation sees such insider know-how as its own form of evidence.

**Fig. 1:**
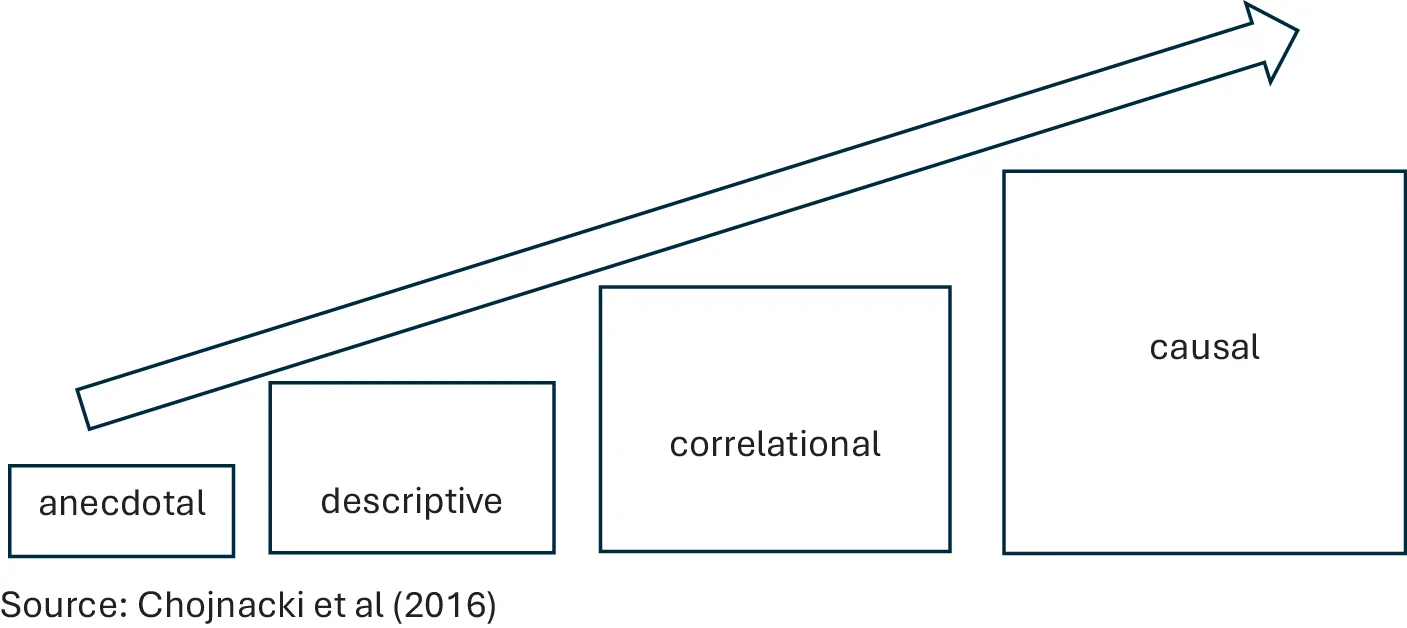
From anecdotal to causal evidence. (Source: Chojnacki et al. 2016)

Other debates target the ‘quality’ of evidence to underpin its suitability for use in EIPM. Chojnacki et al. (2016), working in the field of educational technology, distinguish between four types of evidence and order them in terms of their strength. A visual overview of their work is presented in Fig. 1.

Firstly, ordering their discussion from ‘weak’ to ‘strong’ evidence, they discuss the role of *anecdotal* evidence that is typically generated through accounts of personal experience. In dissemination outputs, it can take the format of testimonials, for example a quote from an adult learner sharing their positive training experiences. Secondly, they present the role of *descriptive* evidence that tends to communicate facts or information but without using them in comparison with other relevant data that might have impacted desired outcomes. When presenting participation rates in ALE (e.g. 60% as ambitioned by the European Commission) without contrasting such data by individual groups or countries, such evidence is ‘descriptive’. Thirdly, Chojnacki et al. (2016) state that *correlational *evidence can allow research users to spot patterns in the data but without the opportunity to claim that one variable is influencing the other or vice versa. This can—for example—be a scatterplot between participation rates in ALE and employment rates in a country. Fourthly, the authors focus on *causal* evidence as the type of evidence that goes beyond presenting correlational patterns in the data. Instead, it allows to make claims on whether the observed effects were truly caused by the introduction of a certain intervention or change in policies or practices. For example, the evaluation of an intervention with jobseekers can reveal whether their participation in a training programme effectively resulted in employment. Such causal claims will even be stronger if such intervention included a control group of jobseekers in a similar situation but who did not participate in the intervention. Chojnacki et al. (2016) assert that causal evidence is the strongest of the four types, given it provides the most rigorous support to judge the potential success of future policy decisions. Such argument suggests that evidence needs to be evaluated and ‘filtered’ before use in decision-making. Baek et al. (2024), however, objected hierarchical approaches to evidence, stating that judgements about evidence need to incorporate questions about its relevance and usefulness for policymaking. As will be discussed below, many modern frameworks on EIPM have already incorporated such critiques, combining the need for methodological rigour with an acknowledgement that decision-making is typically influenced by ‘politics’ and contextual elements too. In this regard, it is important to distinguish between the ‘quality’ of the research underpinning the evidence versus its ‘usability’ for policymaking.

Work specifically on research rigour in the field of education has been taken forward by Gorard et al. (2017, 2020). In their work, robustness is defined in relation to research designs that are fit for purpose to address the relevant research questions, that are sufficiently large scale to allow comparisons between distinct groups, and that are suitable for replication. Robust evidence, they explain, draws on complete data that have minimal missing values to avoid skewed insights and that have minimal attrition across different data collection points in longitudinal research. They argue that a critical look into evidence thus needs to go beyond ‘what’ the evidence is but additionally scrutinise ‘how’ such evidence was generated. Gough’s work—originally applied to systematic reviews—asserts that not all evidence carries the same weight (Gough 2007, 2021). In other words, the ‘Weight of Evidence’ is dependent on the rigour of the underpinning research, the appropriateness and fit of the chosen methods to address the research questions, but also a sound judgment on whether the focus of the evidence gathering is well suited to ensure relevant insights for policy and practice. Such reflections on the reliability, validity and relevance of evidence are important to avoid overclaiming the importance of research results in case their contributions actually ‘weigh rather light’.

### Evidence-use

**Fig. 2:**
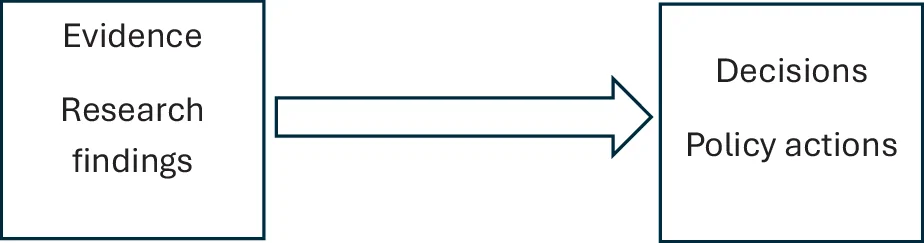
Simplistic representation of the evidence to evidence-use process

In discussing ‘evidence use’, it is essential to avoid taking its purpose for granted. The scholarly literature has presented distinctions between instrumental, conceptual and symbolic uses (Weiss 2021). In simplistic terms, one could assume that researchers produce certain types of evidence, that decision-makers evaluate and filter such evidence for quality, sometimes with the support of knowledge brokers or intermediaries, and then use it to design their policies. In practice, the assessment of evidence might be omitted in real world policymaking (Cairney 2016). Nevertheless, the idea of policymakers translating evidence into specific policy actions to tackle policy problems resembles *instrumental evidence use*. Over the years, however, such ‘linear’ approach from evidence to policy decisions, visualised in Fig. 2, has been criticised for being too simplistic (Best and Holmes 2010). This will be further explained below.

Scholars have argued that the use of evidence is not always targeted towards problem-solving (Malbon and Parkhurst 2023). Rather, *conceptual* evidence-use steers how policymakers think about certain policy problems (Weiss 2021). Such use does not build on a clear understanding of ‘how’ to effectively tackle policy challenges but rather ‘defines’ the policy problem in a certain way. Evidence gathered through survey programmes such as PIAAC provide data against conceptually defined and operationalised measurements of literacy and numeracy skills levels. However, analyses on the cross-sectional dataset will not provide comprehensive and causal evidence on how to increase skills proficiency. Finally, decision-makers can use evidence in a *symbolic* way (Bundi and Trein 2022). As part of this approach, policymakers cherry pick evidence that aligns with their own ideas, with the aim to legitimise their predefined choices. In the field of education, Elfert (2024) pointed at the misuse of evidence to serve decision-makers’ ideologies and own interest. In such situations, the flow presented in Fig. 2 thus gets reversed as the decision-making comes before the selection and filtering of evidence used to justify their policy choices. Weiss (2021) described such evidence-use as tactical and political.

## Models of ‘evidence-informed decision-making’

### From simplistic to integrative models

Apart from the need to understand different types of evidence, it is important to engage with ongoing conceptual advancements on the meanings of and approaches to EIPM. Typically, these have not specifically developed in the field of education or ALE but are reviewed to work towards a model for ALE conceptualisation. As already mentioned above, a ‘linear’ approach from evidence generation to use is now widely perceived as inadequate. Several scholars have investigated the reasons behind the generally limited application of EIPM in public policy and why such ‘linear’ approaches tend to be unsuccessful. For example, Bogenschneider and Corbett (2021) explained through Community Dissonance Theory that decision-making often fails to draw on evidence as researchers and policymakers tend to operate in different workplace cultures. Academic research tends to follow slow timelines because of peer review. Policymakers, on the contrary, aim for faster decision-making and might struggle with complex scholarly jargon. Their professional roles are different, and their institutional environments are dissimilar too: universities and research institutions are distinct from parliamentary offices. There is thus a ‘disconnect’ between the two (Elfert 2024). Cairney (2016) described the ‘bounded rationality’ that underpins policymaking. He argues that decision-makers have limited time and resources to act and cannot entirely separate factual evidence from the norms and values that characterises their work. A fully rational approach to EIPM is therefore unlikely to succeed. Instead, Cairney (2016) advocates for researchers’ increased understanding of how policy processes work as a starting point instead of prioritising an emphasis on ‘evidence’. Similarly, Elfert (2024) states that too much attention to ‘evidence’ can go at the expense of understanding the ‘politics’ of policymaking. For this reason, the term evidence-‘informed’ tends to be used instead of evidence-‘based’ (Head 2016).

Increasingly, the literature argues that relationship building between evidence producers and evidence users is key. Both parties are encouraged to engage with each other throughout the research and decision-making process, potentially supported by intermediaries who specialise in knowledge exchange and transfer. Gough et al. (2018) similarly argue for strengthened engagement between the different actors as a precondition to understand variation in each other’s perspectives and research needs. Research producers and users can support each other to make sense of research findings and to interpret its usability against the social, economic and political contexts in which they operate. The authors label such engagement between them as the backbone of an ‘evidence ecosystem’. This is in line with judgements offered by Baek et al. (2024) that EIPM needs to include reflections on relevance and context to better understand the ‘evidence for whom or what’ question. Such contributions explicitly point towards the need for an integrated model that does not treat ‘evidence’ in isolation from other drivers of decision-making.

Despite ongoing critiques on EIPM, it is important to acknowledge seminal contributions that were made already more than 15 years ago. Best and Holmes’s (2010) *‘Systems thinking, knowledge and action: towards better models and actions’* provided a critical review of traditional Knowledge to Action (KTA) models. Their paper, targeted towards EIPM in health, acknowledges previous conceptualisations that draw on ‘linear’ flows between evidence production and evidence use (resembling Fig. 2), and the importance of ‘relationships’ between stakeholders. Their work mainly aimed to stimulate conceptual integration to arrive at a ‘systems’ approach, acknowledging structural factors that influence the mechanisms of evidence-based decision making. The need to move beyond ‘linear’ models is clearly visible in later work published by scholars such as Cairney (2016), Gough et al. (2018) and Bogenschneider and Corbett (2021). Throughout the last decade, EIPM has thus increasingly integrated the role of policy relevance and the extent to which decision-making is political apart from solely driven by evidence. Newer conceptual models have already attempted to respond to claims that a focus on evidence discards the political and democratic nature of subjects such as education (Biesta 2007; Baek et al. 2024).

### The selection of an integrative model

**Fig. 3:**
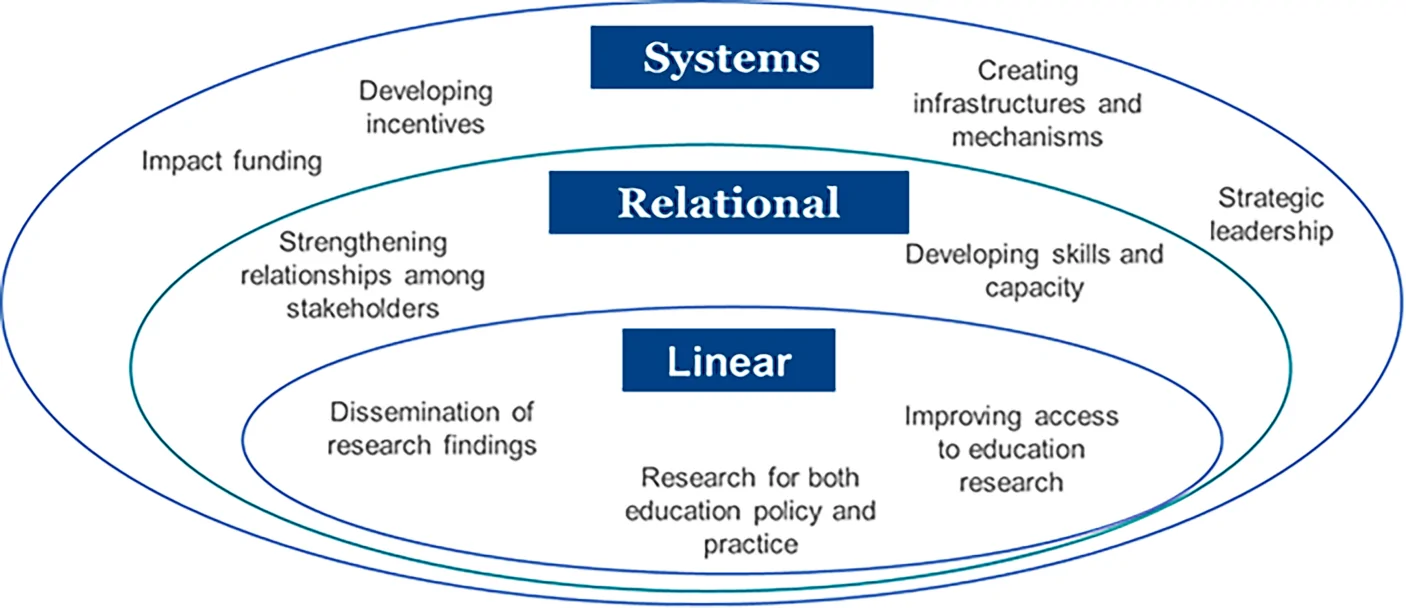
Models of evidence-informed policymaking. (Source: OECD 2024)

While integrative work by Best and Holmes (2010) originated in the field of health, it has been recently used in the wider field of education. Examples include the OECD’s work on ‘Strengthening the Impact of Education Research’ project (OECD 2024). A visual summary as presented in the OECD report is depicted in Fig. 3. As discussed above, the OECD is typically critiqued for its economic and instrumental approach to education. However, it is important to acknowledge the use of Best and Holmes (2010) across a wider range of education pieces by other organisations and authors (Campbell et al. 2017; Maxwell et al. 2022; Rycroft-Smith 2022). In short, these contributions acknowledge the need to integrate linear and relational models to EIPM into a systems approach.

Figure 3 visualises a nested structure that represents a multi-layered ‘logic’, leading to an integrated model. While Best and Holmes (2010) advocated for an expansion of systems approaches, they recognised that it builds on linear and relational approaches too. Instead of discarding these models, they argue to integrate them into a broader systems model.

Put simply, the *Linear Model* underlines the need to disseminate research findings from research producers to research users. Without sharing such research insights, it becomes impossible to use them in EIPM. Research needs to be accessible and transparent to its target audiences, ideally underpinned by FAIR research principles and Open Access availability (Wilkinson et al. 2016). The generation of robust research alone is not sufficient if it does not include efforts to synthesize and disseminate it. On the one hand, research producers can ‘push’ their results towards research users for consideration in their decision-making. On the other hand, research users can ‘pull’ for such evidence by asking for access to research users’ data or findings (Lavis et al. 2003; Bennett and Jessani 2011; Rycroft-Smith 2022). As pointed out above, such use can reveal attempts to use evidence in a symbolic or tactical way (Weiss 2021). In short, to enable EIPM, the ‘linear model’ argues for the generation of evidence but also the uptake of such evidence by decision-makers. This model provides a foundational layer which further models build on but remains too simplistic to accelerate EIPM on its own.

The *Relational Model* attempts to encourage strengthened relationships between relevant stakeholders. This model facilitates the co-creation of knowledge between researchers, practitioners and policymakers and allows them to engage in peer learning across contexts and countries. Best and Holmes (2010) underline the importance of networking based on trust, to work towards common aims and goals, and to understand power dynamics within networks. In working together, the ‘relational model’ incorporates interventions to accelerate stakeholders’ skills, knowledge and attitudes towards EIPM. Previous research observed that evidence uptake can, on the one hand, be constrained by the lack of available or accessible research but, on the other hand, by evidence users’ lack of research literacy, lack of interest or lack of time to engage with and stay-up to date on new research findings (Malin et al. 2025). In Europe, such need for skills and capacity building is recognised within the Framework of the EU Policymaking Competences, part of the Knowledge4Policy project developed by the European Commission’s Joint Research Centre (Dettwiler et al. 2025; Schwendinger et al. 2022). This work strongly advocates for the need for decision-makers to be able to ‘work with evidence’, to ‘be futures literate’ and to be able to ‘collaborate’ and ‘engage with citizens and stakeholders’. Put simply, the ‘relational model’ aims to accelerate skills and capacity to arrive at shared ‘sensemaking’ of evidence by research producers and users.

The *Systems Model*, building on the Linear and Relational Models, explains how evidence use is embedded in its social, economic and political contexts. This model aligns with Cairney’s observations that policymaking processes are complex and that policymakers act within a ‘bounded rationality’ that goes beyond a simplistic use of evidence (Cairney 2016). The ‘systems model’ treats decision-makers as active agents in critical ‘feedback loops’ between knowledge producers and knowledge users, going beyond the ‘relational model’. It embeds them into a wider systems architecture characterised by funding resources, network facilitation, leadership arrangements and incentives to coordinate and implement EIPM. Best and Holmes (2010) see ‘communication’ as a cornerstone to bridge evidence, policy and practice in a complex landscape. This supports processes to arrive at mutual understandings between different actors, and to negotiate potential challenges for decision-making, going beyond simplistic forms of dissemination.

## Advancing the intellectual agenda on EIPM in European ALE

Having selected an integrative model to increase understanding on EIPM, I now turn my attention to ‘sense checking’ the model in the field of ALE. As discussed at the start of this paper, the European Commission wants its Member States to increase the ALE participation rates among their citizens. This is needed, the Commission argues, to fulfil the European Union’s social and economic ambitions, including the twin transition. I use the three models of EIPM (linear, relational, systems, Best and Holmes 2010) to systematically interrogate their potential complexities in the field of European ALE. In doing so, I engage with the relevant ALE literature to unpack ongoing dilemmas in the field to arrive at a better understanding of existing conceptualisation of EIPM in ALE (Jaakkola 2020). As pointed out above, this is important given the strong interest in European ALE policies. However, the conceptual development of EIPM in ALE is lagging in comparison to international organisations’ emphasis on it. I start by mapping complexities against the three models first. I then integrate this into a conceptual framework as a new ‘heuristic device’ to steer the intellectual agenda in the field of EIPM in ALE. As such, it positions itself as a core reference for future studies.

## Complexities

### The linear model

The production of evidence is a foundational precondition to stimulate EIPM (Best and Holmes 2010). While ALE is an active scholarly field, it is necessary to critically unpack its current state to better understand it in the domain of EIPM. In other words, to what extent does the evidence base allow for clear linear dissemination from research users to research producers? Applied to the European policy target of ALE participation, there are several complexities at play.

A first complexity relates to the fuzzy conceptualisation of ‘adult learning and education’ (ALE) (Kalenda and Boeren 2025). The European Commission wants increased participation, regardless of whether adults’ learning takes place in formal or non-formal education and training settings. Interestingly, a few years ago, the Commission decided to discard guided-on-the-job training from the benchmark (Boeren and Kalenda 2025). Informal and everyday learning is also excluded from the 60% target to be achieved by 2030 (OECD 2025). Scholarly work conceptualises ALE through typologies, referring to provision categories such as Adult Basic Education, Adult General Education, Adult Vocational Education, Adult Higher Education and Adult Liberal Education (Desjardins 2017). Both participation in a wine tasting course in a local community education centre (non-formal education) and in an evening degree course for mature students at university (formal education) count towards the European benchmark. Again, this can cause confusion for policymaking and disrupts the ‘linear’ communication between data producers, researchers, wider stakeholders and policymakers. Secondly, recent scholarly work revealed mismatches between Eurostat’s ALE conceptualisation and how such constructs are operationalised for measurement in surveys (Kalenda and Boeren 2025). For example, the European Commission stipulates that participation in formal education needs to represent a minimum of 30 credits under the European Credit Transfer System (ECTS). However, the volume of credits does not get measured in Eurostat data programmes. It makes it harder to compare participation rates in cross-national contexts and leads to ‘noise’ within the communication of evidence to research users. Thirdly, scholars have characterised ALE as a rather homogeneous research field that tends to draw on a select arsenal of methodological techniques, especially qualitative interview studies. Reviews have demonstrated the underrepresentation of experimental, quasi-experimental and causal studies (Boeren 2018; Fejes and Nylander 2019; Nylander et al. 2024; Rubenson and Elfert 2015). As pointed out above though, there is visible objection to the hierarchical treatment of methodologies as it narrows the judgement on what counts as evidence (Baek et al. 2024). Answering those who do put causal design studies at the top of the hierarchy, it is challenging to identify a portfolio of such ALE research. Existing quantitative survey programmes like PIAAC and the Eurostat Adult Education Survey provide cross-sectional data. However, despite being the most niche data sources on participation in ALE, their collection of new data is infrequent, only occurring every five to 10 years. Fourthly, ALE participation data can present birds-eye views on participation patterns, for example by individual background characteristics. However, they lack granularity to appeal to policymakers, limiting the ‘relevance’ of policy messages communicated from research producers to research users. Finally, as in other academic fields, scholarly ALE work typically follows a slower timeline than desirable for policymaking given scholars’ pressures to publish in high impact peer reviewed journals. Despite the increased use of Open Access publishing, many research insights remain hidden behind paywalls. This constrains the ‘accessibility’ and transfer of evidence for broader purposes beyond academic knowledge building. As pointed out above, research producers and research users tend to work in different environments with their own professional cultures (Bogenschneider and Corbett 2021). The need for further interaction and mutual understanding between them will be further unpacked in the section below on the Relational Model.

### The relational model

It is important to disentangle ALE’s ‘relational’ complexity to advance the conceptualisation of EIPM in its field. Firstly, A major constraint for ALE policymaking remains its fragmented landscape. Across countries, policies often require action from decisionmakers in various departments and stakeholders in different sectors (Desjardins and Kalenda 2025; Thunnissen 2023). Research on adult learning systems demonstrates that ALE operates in interaction between ministries of education, labour, economics, social welfare, and health. The fragmentation of the sector can complicate cooperation because of differing views on issues such as budget or curriculum priorities. The provision of ALE is scattered across a wide range of stakeholders, including public and private education and training institutions, workplaces, Public Employment Services, community centres and more. The strong segmentation fuels discrepancies in the understanding of the policy problem. This is especially important against the nature of European ALE policymaking that is strongly steered by the Commission but eventually remains a responsibility of individual Member States (Eeva 2024; Boeren 2025). Secondly, and interrelated, our own research in the UK context revealed examples of frictions between policymakers and stakeholders active in other segments of the ALE sector (Boeren & Clancy, forthcoming). For example, colleagues in trade unions tended to feel ignored by the Conservative government despite their willingness to build relations and provide feedback on policies. Trust and mutual understanding between different actors were absent despite such elements being of central importance in the ‘relational model’. It raises questions about who is included or excluded from the ‘relational model’ and what drives such representations. Thirdly, the ALE sector struggles with rapid staff change over in policy departments, leading to loss of institutional memory (Flemons et al. 2024). Within a ‘relational model’, networks are not one-off situations but rather require continuation over time with iterative feedback on policies flowing between them. Fourthly, and interconnected to the third point, this raises further concerns about decisionmakers’ knowledge and understanding of the evidence base on ALE, and evidence producers’ grasp of policymaking processes (Bogenschneider and Corbett 2021). Projects like the European Commission’s Knowledge4Policy were established to enhance competencies for policymaking, both among researchers and decision-makers (Schwendinger et al. 2022). It is unclear to what extent Knowledge4Policy toolkits on EIPM policymaking are used by ALE stakeholders. While not specifically designed for the field of ALE, there is no ‘evidence’ to suggest that the European ALE sector does not experience similar obstacles in aligning evidence with policymaking compared to other policy domains.

### The systems model

While international organisations such as the OECD and the European Commission have invested in projects to accelerate EIPM, there is currently no overarching governance framework to enable this in the field of ALE. This is a first complexity that needs to be understood to advance conceptualisation on EIPM in European ALE. It raises questions on who exactly needs to be included in leadership for EIPM and where the coordination for it should sit. Secondly, systematic benchmarking of the European ALE participation target has continued to cause confusion given the multitude of data sources, fragmenting the evidence base (Kalenda and Boeren 2025). As pointed out earlier in this paper, the Commission had to produce technical notes to clarify its calculation of ALE participation statistics given growing criticism on its weaknesses. It demonstrates how evidence production for policy purposes requires strategic leadership and systemic opportunities for feedback loops between actors. Again, this is especially important against the nature of European ALE policy that remains a core responsibility of the Member State (Boeren 2025). Thirdly, as discussed earlier in this paper and critiqued in the scholarly literature, the current approach to knowledge gathering on ALE participation is mainly steered by the OECD and the European Commission itself. Researchers have access to relevant ALE data through PIAAC’s Public Use Files or through application for Eurostat data, including the Adult Education Survey, Labour Force Survey and Continuing Vocational Education and Training Survey. The Consortium of European Social Sciences Data Archives hosts a wider catalogue of secondary data, including interview transcripts or qualitative data input from previously completed research studies (Late and Kekäläinen 2020). However, this is far from a comprehensive overview of the existing evidence as it does not yet succeed in absorbing wider sources of knowledge as defined under a broader conceptualisation of ‘evidence’. While leadership in a systems model can help evidence to be produced and actioned on, it also tends to steer what types of evidence gathering get prioritised. Fourthly, this relates to how resources and incentives for EIPM get distributed among relevant actors in the systems model. For example, the European Commission coordinates programmes like Horizon Europe to enable the collection of new evidence, the analysis of secondary data and the design of pathways to policy impact. Calls for European Research Council Grants and Marie Curie fellowships and doctoral training centres are launched annually, exemplifying a systems approach to incentivising research production and the stimulation of its use among stakeholders. However, such grant schemes are highly competitive, leading to a low volume of such funding flowing to ALE projects. Fifthly, the systems model can further enable policy impact and uptake through supporting a culture of Open Access of research outputs and research data. Accessibility limits a ‘linear’ approach to EIPM but can be reduced to systemic investments.

## Towards an integrative conceptualisation

**Fig. 4:**
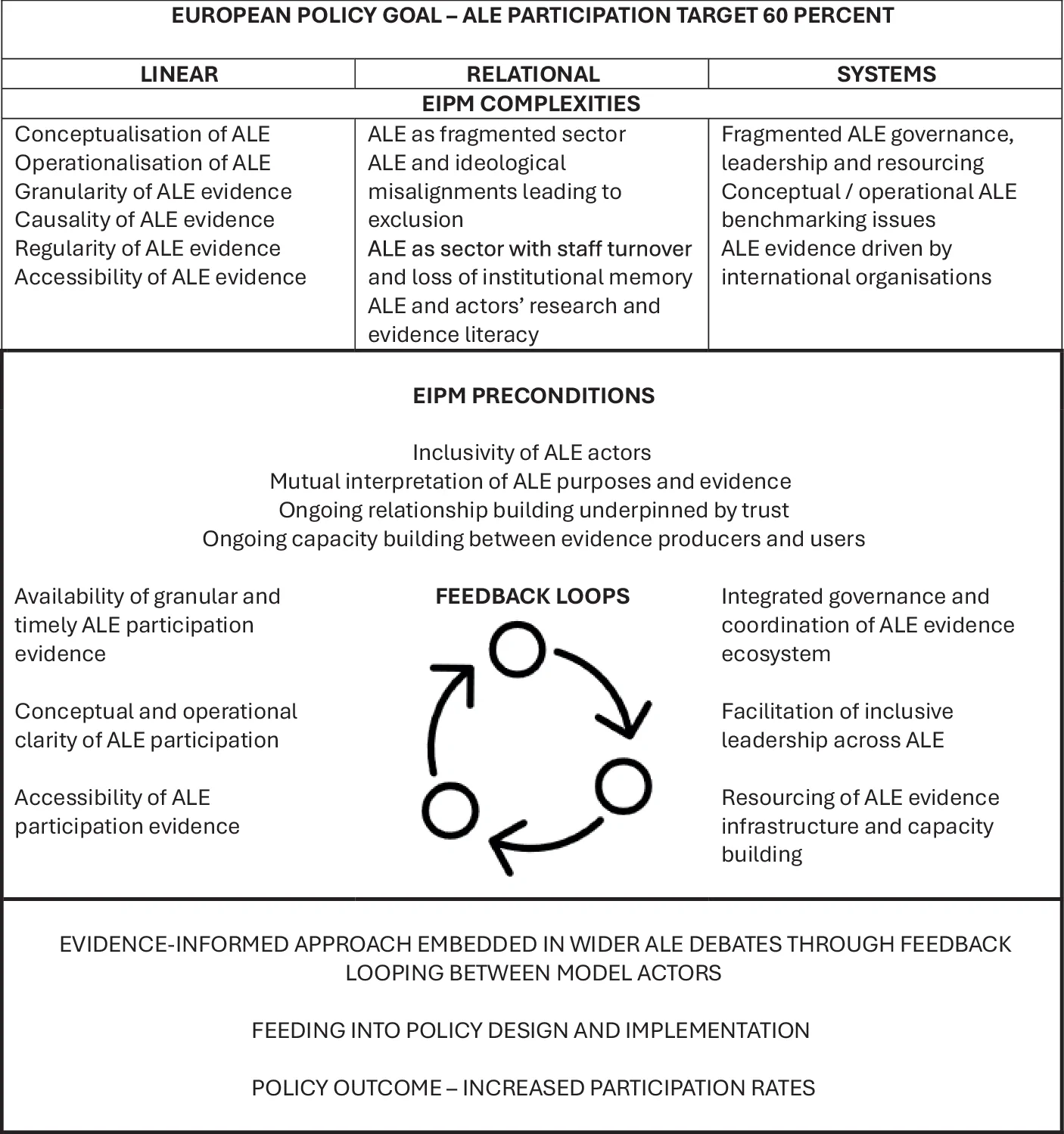
Conceptual framework as heuristic device to understand EIPM in relation to European policy on ALE participation

So far, I have discussed the conceptual evolution of EIPM. In doing so, I have uncovered the complexities in understanding this approach in relation to the European Commission’s policy ambitions to increase ALE participation rates across its Member States. Drawing on Best and Holmes (2010), I organised these observations according to Linear, Relational and Systems Models of EIPM. In Fig. 4, I visually summarise these complexities. However, I go one step further to build a ‘heuristic device’ that offers an integrated conceptualisation of EIPM’s preconditions to advance participation in ALE in the context of European policies (Jaakkola 2020). These preconditions correspond to ongoing complexities on the conceptualisation and operationalisation of ALE, the need for more granular, timely and accessible evidence. It conceptualises the role of ALE actors through inclusive and sustainable relationships underpinned by trust and positions systemic coordination and cooperation against ALE’s ongoing sectoral and policy fragmentation. The central feature of Fig. 4 represents ‘feedback looping’ to integrate different conceptual models into a framework that aims to understand how evidence is framed, gathered, mobilised and actioned between all relevant ALE actors. This is important as ALE spans the boundaries of different policy domains and requires intersectoral as well as cross-national cooperation. These ‘feedback loops’ answer frequent critiques that EIPM needs to balance its focus on ‘evidence’ with a deeper understanding of further drivers of policymaking. These include the politics of policymaking, the role of institutions and governance approaches. An integration through ‘feedback looping’ is conceptualised to boost mutual understanding and interpretation of ALE purposes and the potential role of evidence in policymaking. Eventually, in theory, such embedded approach could then steer the design and implementation of new policies to accelerate participation in ALE across the European Member States.

## Conclusions

This paper integrated insights from the literature on evidence and evidence use in policymaking with the European Commission’s policy ambition on participation in ALE. It outlined the conceptual evolution of EIPM to answer criticisms on narrow definitions of what counts as evidence, who is included in decision-making, and to what extent policymaking is driven by politics rather than research (Best and Holmes 2010). It used Linear, Relational and Systems Models to uncover complexities of EIPM in ALE and conceptualised EIPM preconditions to better understand the nature of EIPM in relation to the European ALE participation target. Rather than a prescriptive framework on EIPM, I contributed a conceptual heuristic device as a core reference to inform the intellectual agenda on an under-researched policy approach in the field. I welcome future research to refine this conceptualisation and to empirically investigate the role of ‘feedback looping’ to better understand the role of evidence in European ALE policymaking. This could start with a mapping exercise on the extent to which evidence—and what types of evidence—is currently drawn upon in policy documents. This could be combined with a comprehensive stakeholder mapping to uncover contributors to ALE policymaking across Europe. Research could trace policies from initial ideation to implementation to better understand the role of evidence. In doing so, it could zoom in on actor involvement and the processes of feedback looping between them. Research could even bring together actors as study participants to simulate their approaches to EIPM in a scenario-based format. Given the complex nature of ALE, future research should ideally pay attention to questions of leadership, governance and resourcing across ALE sectors and policy domains. It should also incorporate a cross-country comparative element to account for the fact that policy responsibility for ALE participation remains at the national level.
